# Photothermally Responsive Conjugated Polymeric Singlet Oxygen Carrier for Phase Change-Controlled and Sustainable Phototherapy for Hypoxic Tumor

**DOI:** 10.34133/2020/5351848

**Published:** 2020-10-10

**Authors:** Guo Li, Ruyi Zhou, Weili Zhao, Bo Yu, Jie Zhou, Shujuan Liu, Wei Huang, Qiang Zhao

**Affiliations:** ^1^Key Laboratory for Organic Electronics and Information Displays & Jiangsu Key Laboratory for Biosensors, Institute of Advanced Materials (IAM), Nanjing University of Posts and Telecommunications (NUPT), 9 Wenyuan Road, Nanjing, 210023 Jiangsu, China; ^2^Frontiers Science Center for Flexible Electronics, Xi'an Institute of Flexible Electronics (IFE) and Xi'an Institute of Biomedical Materials & Engineering, Northwestern Polytechnical University, 127 West Youyi Road, Xi'an 710072, China

## Abstract

Hypoxia significantly compromises the therapeutic performance of photodynamic therapy (PDT) owing to the oxygen level which plays a key role in the production of singlet oxygen (^1^O_2_). Herein, the photothermally responsive phase change materials (PCM) are used to encapsulate 1,4-dimethylnaphthalene-functionalized platinum(II)-acetylide conjugated polymer (CP1) with intense near-infrared (NIR) absorption to prepare new ^1^O_2_ nanocarriers (CP1-NCs). The 1,4-dimethylnaphthalene moieties in CP1-NCs can trap the ^1^O_2_ produced from CP1 under irradiation and form a stable endoperoxide. Then, the endoperoxide undergoes cycloreversion to controllably release ^1^O_2_ via the NIR light-triggered photothermal effect of CP1 and controllable phase change of PCM, which can be used for oxygen-independent PDT for hypoxic tumor. Furthermore, the *in vivo* luminescence imaging-guided synergistic PDT and photothermal therapy showed better efficiency in tumor ablation. The smart design shows the potent promise of CP1-NCs in PCM-controlled and sustainable phototherapy under tumor hypoxic microenvironment, providing new insights for constructing oxygen-independent precise cancer phototherapeutic platform.

## 1. Introduction

Photodynamic therapy (PDT), as a well-known and emerging clinical treatment solution, can transform oxygen (O_2_) into toxic reactive oxygen species (ROS) to eradicate cancer cells [1–4]. As a consequence, considerable efforts have been devoted to enhancing PDT due to their minimal invasiveness, high selectivity, accurate spatiotemporal regulation, and minimized side effect [5–9]. So far, a large majority of reported PDT therapeutic systems depend highly on O_2_ level [10–14]. However, the consumption of O_2_ in PDT process and rapid proliferation of tumor cells aggravate hypoxic microenvironments [15–17]. Thus, tumor hypoxia is traditionally considered the “Achilles' heel” of PDT, which not only accelerates metastasis in many solid tumor cells but also significantly causes therapy resistance and low antitumor efficiency [18–23].

To date, various innovative strategies have been extensively utilized to relieve tumor hypoxia and enhance the therapeutic effect of PDT [18, 20, 21]. One is to integrate PDT with chemotherapy for synergistic therapy [24–26]. However, such chemophototherapeutic prodrugs are complex multicomponent nanosystems, which often lead to the difficulty in the preparation and suffer from the threat of release of drug in normal cells and tissues. Another approach is to directly transport O_2_ into tumor sites through oxygen-carrier nanomaterials, including perfluorocarbon, CaO_2_, MnO_2_, carbon nitride, enzymes, or oxygenated hemoglobin [27–30]. The enhanced PDT efficiency could be achieved by the quantitative O_2_ generation performance. However, in these oxygen self-supplying nanomaterials, there remain many severe challenges, including unexpected side effects and unpredictable toxicity in the complicated physiological environment. In addition, many smart phototherapy platforms for in situ generation of hydroxyl radicals within tumors based on the unique tumor microenvironment feature through Fenton reaction have been developed, which possess high therapeutic specificity and low invasiveness [31–36]. However, most of these nanoplatforms are also based on complex multicomponent nanosystems [27–31]. Although many prominent works have been developed, there is an urgent need to construct a new PDT therapeutic system for overcoming the tumor hypoxia and achieving sustainable phototherapy.

**Figure 1:**
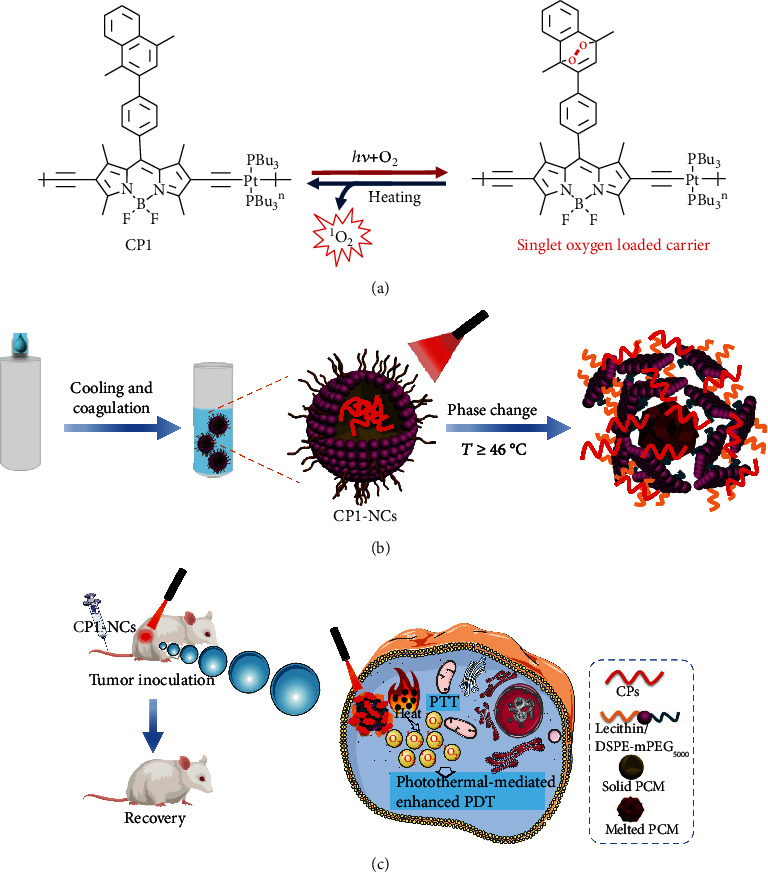
(a) Mechanism illustration of capture and release of ^1^O_2_ by the CP1 as ^1^O_2_ carrier. (b) The CP1-NC construction through the modified nanoprecipitation and the photothermal-triggered release of CP1-NCs and PCM. (c) The responsive process of controlled and sustainable phototherapy under hypoxia.

Herein, we developed a novel photothermally responsive conjugated polymeric singlet oxygen (^1^O_2_) carrier, achieving phase change-controlled and luminescence imaging-guided sustainable phototherapy for hypoxic tumor (Figure 1). Platinum(II)-acetylide conjugated polymers (CPs) containing boron dipyrromethene (BDP) units with intense near-infrared (NIR) absorption were chosen as a perfect phototherapeutic platform [37–44], which simultaneously possess PDT and photothermal therapy (PTT) properties. 1,4-Dimethylnaphthalene, as the efficient ^1^O_2_ carrier, was introduced into CPs (CP1), which can reversibly trap and photothermally release ^1^O_2_ (Figure 1(a)), providing a new strategy for directly delivering ^1^O_2_ to relieve hypoxic tumor [45–47]. As compared to many other stimulus-responsive platform, photothermally responsive nanoplatform via phase change materials (PCM) with a large potential fusion heat and reversible solid-liquid phase change in a relative narrow temperature region is more appropriate for accurately controlled release [48–51]. Therefore, the CP1 nanocarriers (CP1-NCs) were prepared through coencapsulating hydrophobic CP1 in biocompatible organic PCM (a mixture of oleic acid and hexadecanol, melting point at 46°C) with amphiphilic lecithin and DSPE-mPEG_5000_. Meanwhile, the novel CP1-NCs were injected into a tumor-bearing mouse via the tail vein and effectively accumulated in tumor location via the improved permeability and retention (EPR) effect [52, 53]. Besides, the CP1-NCs could not only act as a NIR luminescence imaging contrast agent but also trap the ^1^O_2_ produced from CP1 under irradiation and form a stable endoperoxide (EPO). Furthermore, the EPO undergoes cycloreversion to controllably release ^1^O_2_ via the NIR light-induced photothermal effect of CP1 and controlled phase change of PCM, which can be used for oxygen-independent PDT for hypoxic tumor. Therefore, the new CP1-NC therapeutic nanoplatform could successfully relieve tumor hypoxia for oxygen-independent sustainable phototherapy and provide valuable guideline to develop high-performance theranostic agents in cancer treatment.

## 2. Results and Discussion

### 2.1. Synthesis and Characterization

BDP-based CPs (CP1-CP3) were synthesized from the BDP precursor and Pt1 or 1,4-diiodobenzene through dehydrohalogenation reaction [54, 55]. The monomer *trans*-dichlorobis (tri-n-butylphosphine) platinum(II) (Pt1) was obtained from the reported methods [56–58]. The detailed synthetic routes of BDP precursor and CP1-CP3 are shown in Figures S1 and S2. CP2 and CP3 without Pt center and 1,4-dimethylnaphthalene-functionalized group were designed as the control. CP1-CP3 were purified through silica column chromatography and precipitated in methanol, generating the dark green and purple polymers. The ^1^H NMR, ^13^C NMR, and matrix-assisted laser desorption/ionization time-of-flight mass spectrometry were used to characterize the purity of intermediates and CPs. Furthermore, the weight average molecular mass (*M*_w_) of CP1, CP2, and CP3 was 9500, 8500, and 8500 with polydispersity indexes (PDI) of 1.11, 1.09, and 1.14, respectively.

**Figure 2:**
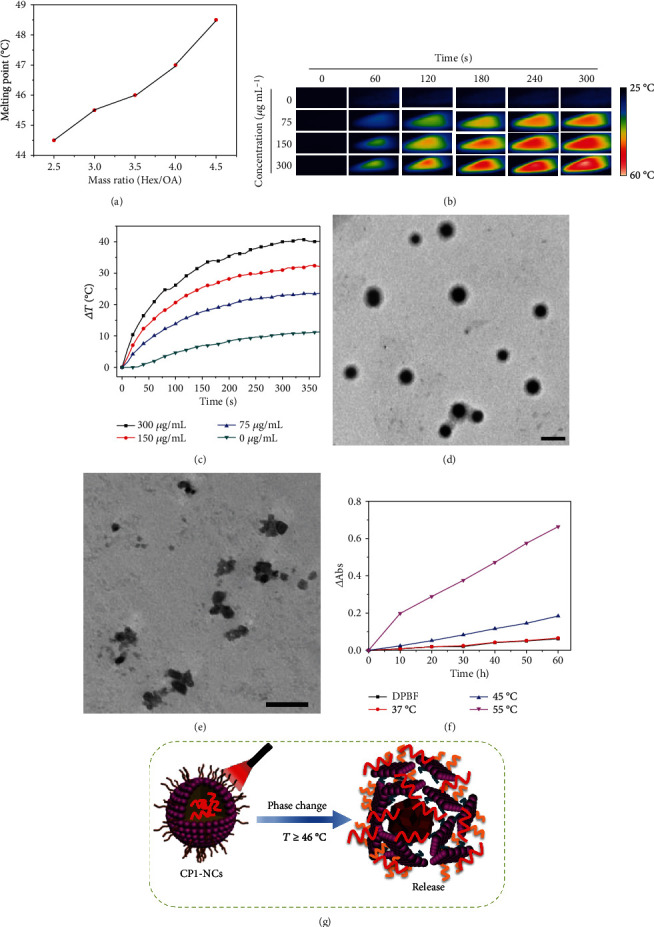
(a) Melting point change of PCM with different Hex/OA mass ratios. (b) Photothermal images of CP1-NCs. (c) Temperature elevation of CP1-NCs at different levels under 690 nm light irradiation at 0.5 W cm^−2^ for 6 min. TEM of CP1-NCs, (d) before, and (e) after irradiation for 6 min. Scale bars: 200 nm. (f) ΔAbs of DPBF under various temperatures in the mixture solution of CP1-NCs and DBPF. (g) Schematic illustration of photothermal-induced release of CP1-NCs.

**Figure 3:**
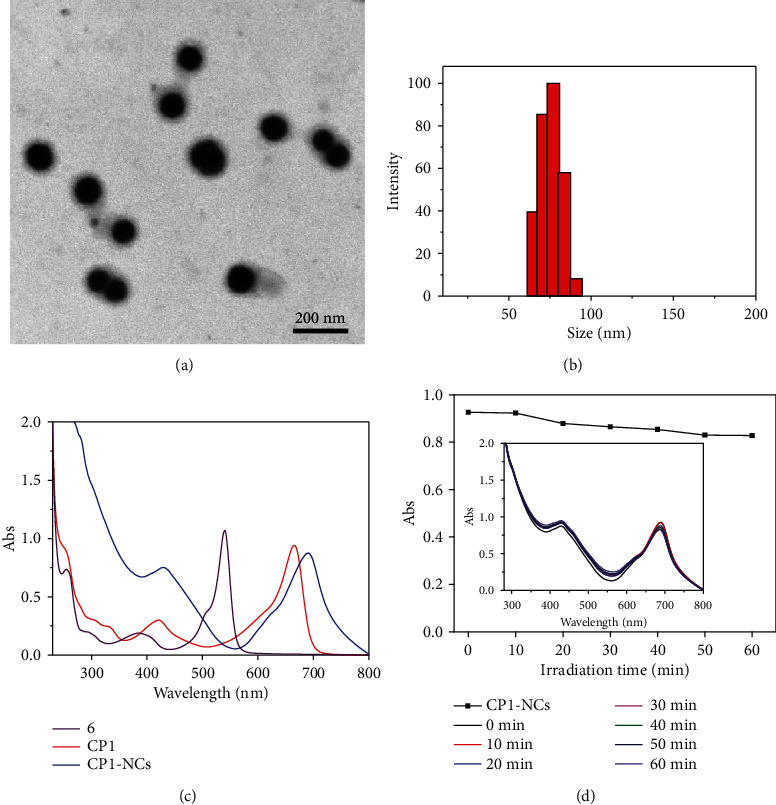
(a) TEM imaging of CP1-NCs. (b) DLS of CP1-NCs. (c) Absorption spectra of 6, CP1, and CP1-NCs. (d) Photostability of CP1-NCs.

As shown in Figure 1(b), the CP1-NC formation was illustrated. The PCM were prepared according to the reported procedures [48]. Through modulating the mass ratio of oleic acid (OA) and 1-hexadecanol (Hex) at 1 : 3.5, PCM with a melting point of 46°C were obtained (Figure 2(a)). Through coencapsulating hydrophobic CP1-CP3 into PCM with amphiphilic lecithin and DSPE-mPEG_5000_, the water-soluble CP1-NC and CP nanoparticles (CP-NPs) were acquired. The concentrations of CP1-NCs and CP-NPs were calculated according to the absorption of Figure S4. The CP1-NCs were fully characterized by transmission electron microscopy (TEM) and dynamic light scattering (DLS). TEM imaging showed that CP1-NCs possessed a uniform morphology, revealing the diameter about 35 nm (Figure 3(a)). DLS indicated that CP1-NCs possessed an average hydrodynamic size of around 78 nm (Figure 3(b)), suggesting the potential passive tumor targeting ability via EPR effect [52].

### 2.2. Photophysical Properties

The photophysical properties of CPs, CP1-NCs, and CP-NPs were explored by the absorption and photoluminescence (PL) spectra. As exhibited in Figures 3(c) and S5, the maximal absorption peaks of CP1-CP3 were located at 660, 575, and 650 nm in CH_2_Cl_2_, respectively, which are assigned to the *π*‐*π*∗ transition of the BDP platinum-acetylide backbone, showing significant red shift in comparison with that of their corresponding BDP precursor owing to the *π*-electron delocalization and rigid planar structures [42, 59]. The absorption spectra of CP1-NCs displayed a strong absorption band at 254 nm, which was consistent with the absorption of CP1 and 6. After the coordination with BDP precursor 6 or 7, CP1-CP3 possessed intense NIR emission in the region of 650-750 nm (Figure S6). Compared with the CPs, the formed CP-NPs showed significant suppression of the luminescence, which endows a high potential for CP-NPs as a photothermal agent [38, 60]. The strong *π*‐*π* stacking in the micellar cores leads to the quenching of fluorescence [60]. Furthermore, photostability is the key in cancer phototherapy, and the resistance of CP1-NCs to photobleaching was measured. The absorption of CP1-NCs showed no significant change during 60 min under 690 nm light irradiation, demonstrating the excellent antiphotobleaching performance (Figure 3(d)).

### 2.3. Photodynamic and Photothermal Properties

The photothermal effect of CP1-NCs plays an important role in the process of triggering ^1^O_2_ release. Therefore, the temperature elevation of CP1-NCs was firstly explored at various concentrations under 690 nm irradiation (0.5 W cm^−2^). CP1-NCs showed remarkable temperature elevation from 21 to 39°C with the increasing of concentration, which was much higher than that of the control CP2-NPs (Δ*T* ≈ 9°C) (Figures 2(c) and S8). The remarkable temperature elevation of CP1-NCs demonstrates that the introduction of Pt can enhance the photothermal efficiency through the improved nonradiative decay process [38]. Besides, the photothermal conversion effect of CP1-NCs was tested by temperature elevation through a cycle of heat-up and cooling after cessation of light irradiation. CP1-NCs showed the superior photothermal conversion effect of 49.0% compared with the existing photothermal materials such as aza-boron-dipyrromethene dye nanoparticles (43.0%), Pt-based nanoparticles (37.0%), semiconducting polymer nanoparticles (26.7%), cyanine dye nanocarriers (26.6%), and gold rods (21.0%) [38, 61–64]. Moreover, CP1-NCs exhibited almost no large difference in their temperature monitoring after five irradiation/cooling cycles (Figure S8(a)), suggesting their superior resistance to photobleaching under irradiation.

Then, we explored the morphological changes of the CP-NCs before and after 690 nm laser irradiation. TEM imaging suggested that light irradiation generated significant influence not only on the particle size but also on the morphology of the CP1-NCs@PCM (Figures 2(d) and 2(e)). After irradiation, the morphological change of CP1-NCs was observed from uniform sphere to amorphous structure by TEM imaging. And the hydrodynamic diameters of the nanocarriers became smaller (from 74 ± 6 nm to 36 ± 5 nm) through DLS (Figure S3). For CP2-NPs, no significant difference was found in the particle size and morphology of the CP2-NPs@PCM after irradiation, which was attributed to the negligible photothermal efficiency of CP2-NPs.

Afterwards, the sustainable PDT property of CP1-NCs was demonstrated through investigating the ^1^O_2_ trap and release process. The ^1^O_2_ capture capability of CP1-NCs was investigated through the absorption decrease due to the EPO formation. Along with the increase of irradiation time, the absorption band at 254 nm decreased gradually, which demonstrated that ^1^O_2_ sensitized by CP1-NCs was captured by itself to generate EPO (Figure S7). Under 690 nm irradiation, the CP1-NCs were able to sensitize oxygen to produce ^1^O_2_, which displayed a decreasing in absorbance at 420 nm (ΔAbs) in the presence of ^1^O_2_ by 1,3-diphenylisobenzofuran (DPBF) as an indicator (Figure S7(a)). Furthermore, the ^1^O_2_ release of CP1-NCs was carried out under dark conditions at three temperatures of 37, 45, and 60°C, respectively. As exhibited in Figure 2(f), a higher temperature could accelerate the release of ^1^O_2_. In contrast, when 1,4-dimethylnaphthalene-free CP3-NPs were irradiated, the negligible ΔAbs values were observed (Figure S7(b)). The results suggested that CP1-NCs not only could effectively form EPO under irradiation but also release ^1^O_2_ by the photothermal effect in aqueous solution. As shown in Figure 2(g), a hypothetical model further illustrated photothermal-induced release of CP1-NC under irradiation. Especially, compared to traditional photosensitizer, O_2_ is unnecessary in ^1^O_2_ release process of CP1-NCs, suggesting great potential for enhancing the phototherapeutic performances of hypoxia-associative PDT. Therefore, the excellent PDT/PTT performances of CP1-NCs endowed it as potential phototherapy agents for the following NIR light-induced therapy experiments.

### 2.4. Cytotoxicity Assay and PDT/PTT Efficiency of CP1-NCs

**Figure 4:**
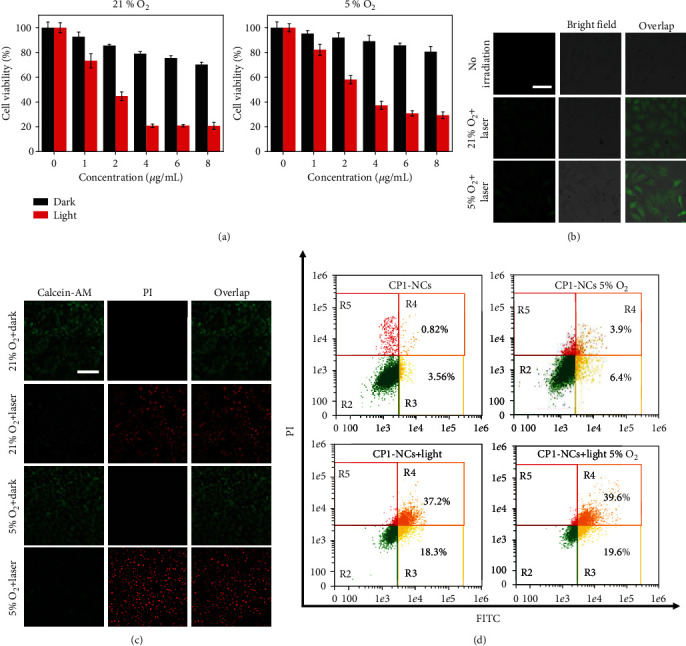
*In vitro* evaluation of CP1-NCs under 21% or 5% oxygen concentration. (a) MTT assay of CP1-NCs with and without irradiation. (b) Confocal image of HeLa cells cultured with CP1-NCs with or without 5 min light irradiation (690 nm, 0.5 W cm^−2^) using DCFH-DA staining (*λ*_ex_ = 488 nm, *λ*_em_ = 500‐540 nm, scale bar: 100 *μ*m). (c) Calcein AM and PI-stained HeLa tumor cells treated with CP1-NCs with or without 5 min light irradiation (690 nm, 0.5 W cm^−2^, scale bar: 100 *μ*m). (d) Flow cytometry quantification of apoptosis of HeLa cells incubated with CP1-NCs under 690 nm light irradiation (0.5 W cm^−2^).

The cytotoxicity of CP1-NCs *in vitro* was explored through the 3-(4,5-dimethyl-2-thiazolyl)-2,5-diphenyl-2H-tetrazolium (MTT) assessment. HeLa cells were treated with CP1-NCs at various concentrations (1.0, 2.0, 4.0, 6.0, and 8.0 *μ*g mL^−1^) for 24 h and then conducted with 690 nm light irradiation under 21% and 5% O_2_ for 6 min or not, respectively (Figure 4(a)). The HeLa cell viability in the absence of irradiation was larger than 77% at the level of 8.0 *μ*g mL^−1^, demonstrating the outstanding biocompatibility of CP1-NCs. Furthermore, the cells cultured with ^1^O_2_ loading CP1-NCs displayed relatively lower cell viability under 21% or 5% O_2_ concentration, showing that the CP1-NCs could trigger cell oxidative damage and kill cells even under hypoxia condition. Owing to the intrinsic NIR emission of CP1-NCs, the cellular uptake of CP1-NCs was further explored. The red fluorescence in the cytoplasm was found from the cells cultured with CP1-NCs through the confocal images, indicating the abundant uptake and excellent dispersion of CP1-NCs in HeLa cells (Figure S9). To investigate the phototherapy performance of the CP1-NCs including ^1^O_2_ generation, 2′,7′-dichlorodihydrofluorescein diacetate (DCFH-DA) [65], which could be transformed into DCF with green luminescence in the presence of ^1^O_2_, was used as a ^1^O_2_ indicator. As exhibited in Figure 4(b), the strong green luminescence was observed from the HeLa cells treated with CP1-NCs under 21% and 5% O_2_ level, illustrating the production of abundant intracellular ^1^O_2_ under irradiation. Moreover, the CP1-NCs generated negligible fluorescence in the absence of irradiation, since the physiological temperature (around 37°C) was rather low so that it might not induce the release of ^1^O_2_ from EPO. The results suggested that the release of ^1^O_2_ from EPO might be triggered through the photothermal effect of CP1-NCs.

To explore the *in vitro* cell killing performance, calcein AM (live cells) and propidium iodide (dead cells) staining assessments were carried out under normoxia and hypoxia. When treated with CP1-NCs, the cells were dead under either 21% or 5% oxygen level with irradiation (Figure 4(c)). In the absence of light irradiation, the HeLa cells incubated with CP1-NCs mainly maintained alive under 21% and 5% oxygen levels. The results confirmed that the phototherapeutic performance was unaffected by the hypoxia. In addition, to demonstrate the cell population at different periods of apoptosis, flow cytometry assay was explored (Figures 4(d) and S10). Under 690 nm light irradiation, the significant improved cells were achieved in the fluorescein isothiocyanate- (FITC-) positive and PI-positive area under normoxia and hypoxia. The results demonstrated that the photothermal efficiency of CP1-NCs induced the release of ^1^O_2_ and oxidative damage improved the phototherapeutic performances, motivating us to explore the application of CP1-NCs for enhanced tumor phototherapy.

### 2.5. *In Vivo* Luminescence Imaging, Photothermal Imaging, and DCFH-DA Staining of CP1-NCs

**Figure 5:**
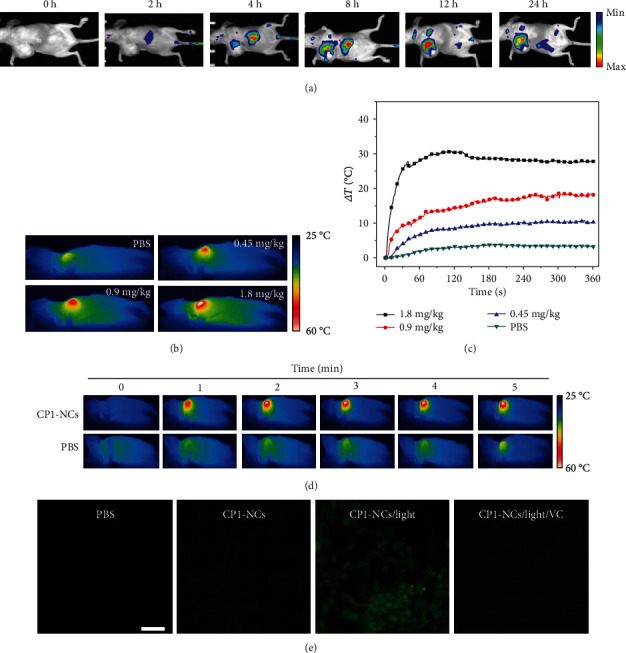
*In vivo* photothermal performances and production of singlet oxygen of CP1-NCs. (a) Luminescence image of the HeLa tumor-bearing mice at various time points through tail intravenous injection of CP1-NCs (*n* = 3 mice per group). (b) Infrared thermograph of the mice bearing HeLa tumor treated with CP1-NCs at different concentrations under 690 nm irradiation at 0.5 W cm^−2^ for 6 min and (c) the corresponding temperature elevation within tumor region. (d) Representative thermal images of mice (tumor sites) subjected to 690 nm light irradiation for 12 h postinjection of CP1-NCs (0.9 mg kg^−1^, 120 *μ*L) and PBS. (e) DCFH-DA staining of tumor slice from the mice treated with CP1-NCs at 12 h postinjection in the absence or presence of VC under light irradiation or not (scale bar: 100 *μ*m).

According to the NIR emission of CP1-NCs, the *in vivo* luminescence imaging of CP1-NCs was used to acquire the accurate therapeutic time. The CP1-NCs were intravenously injected into the tumor-bearing mice, and their biodistributions were recorded at various postinjection time points. The luminescence signal in the tumor sites quickly increased from 2 h to 12 h after injection and decreased as the metabolism of CP1-NCs (Figure 5(a)). The signal intensity reached to its plateau at 12 h postinjection, which was acted as the reference time for the following therapy. In comparison, negligible or weak luminescence intensity was showed in other main organs (Figure S11). These imaging results suggested superior ability of the CP1-NCs to accumulate in tumor location, which was attributed to the EPR effect via the proper particle size.

To explore the performance of CP1-NCs to produce hyperthermia *in vivo*, CP1-NCs were injected into the mice at different doses, followed through infrared thermal imaging of mice after 12 h injection under 690 nm irradiation (0.5 W cm^−2^). PBS, as a control, led to a negligible temperature increase (Figure 5(b)). Under 690 nm irradiation, CP1-NCs at the doses of 0.45, 0.9, and 1.8 mg kg^−1^ CP1 triggered the tumor temperature changes of 11, 19, and 30°C, respectively, suggesting remarkable temperature elevations with the increasing of dose (Figures 5(b)–5(d)). Because too high temperature increase produced detrimental influence for the normal cell, and too low temperature cannot obtain appropriate photothermal therapy efficiency, the level of CP1-NCs at 0.9 mg kg^−1^ CP1 was selected for the subsequent tumor therapy. Furthermore, the capability of CP1-NCs producing ROS at the tumor slice was investigated by the DCFH-DA staining under 690 nm light irradiation. Vitamin C (VC) as a ^1^O_2_ scavenger was used to scavenge ^1^O_2_. As displayed in Figure 5(e), the strong green luminescence was found at the tumor slice of mice cultured with CP1-NCs under light irradiation. However, the HeLa cells with CP1-NCs without light irradiation or in the presence of VC induced negligible green luminescence. The results suggested that CP1-NCs could generate abundant ^1^O_2_ under irradiation.

### 2.6. Synergistic Phototherapy

**Figure 6:**
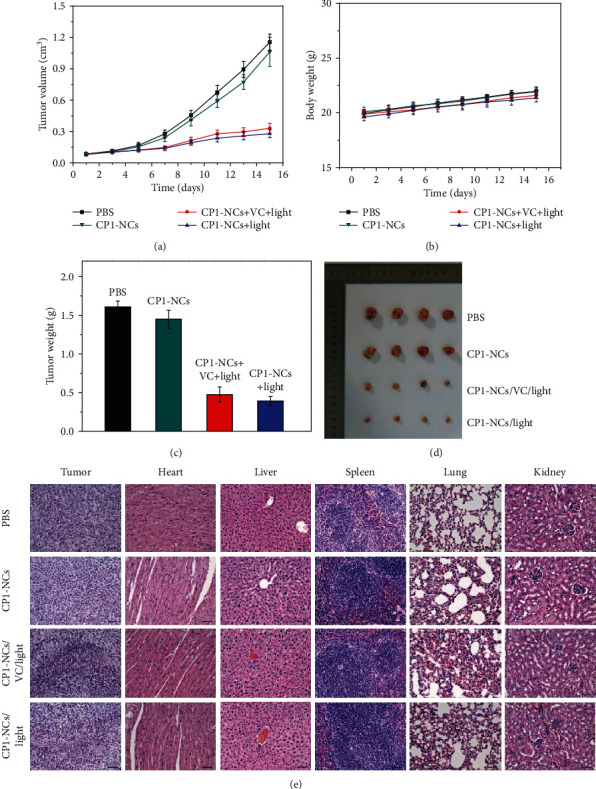
(a) Relative tumor volume changes of mice with various therapies. (b) Body weight changes of mice with different therapies. (c) Tumor weight of mice with different treatments. (d) Photos of each group of mice after the treatment. (e) H&E staining of the tumor, heart, liver, spleen, lung, and kidney obtained from the tumor-bearing mice with different treatment groups (scale bar: 100 *μ*m).

Encouraged by the preferable luminescence imaging, sustainable ^1^O_2_ production, and good photothermal conversion efficiency of CP1-NCs, the phototherapy effect of CP1-NCs was carried out *in vivo* using the HeLa tumor mouse model. After the tumor volume increased around 120 mm^3^, the mice were separated into four groups. The mice were treated with (I) PBS, (II) CP1-NCs only, (III) CP1-NCs (150 *μ*L, 0.9 mg kg^−1^ CP1)+irradiation (690 nm, 0.5 W cm^−2^)+VC, and (IV) CP1-NCs (150 *μ*L, 0.9 mg kg^−1^ CP1)+irradiation (690 nm, 0.5 W cm^−2^), respectively. The fourth group acted as the therapy group. The I-III groups served as control. The mouse tumor volume and weight were recorded every two days. For groups (III) and (IV), 6 min irradiation was implemented after 12 h injection. As exhibited in Figure 6(a), the tumor volumes of (I) and (II) exhibited an expeditious growth in the absence of irradiation, demonstrating the excellent biocompatibility of CP1-NCs. Compared with tumors treated with CP1-NCs+irradiation+VC, the tumor was significantly inhibited for those treated with CP1-NCs+irradiation, suggesting that the continuous delivery of ^1^O_2_ leads to better phototherapy performance. Similar body weight change was observed in the control groups in comparison with the treatment group, suggesting the negligible side effects of CP1-NCs (Figure 6(b)). The photo of tumors was consistent with the monitored tumor weight (Figures 6(c) and 6(d)). The therapy results confirmed that CP1-NCs can achieve enhanced PDT performance through the ^1^O_2_ release for inhibiting tumor growth, which could act as a potential phototherapy agent for precision tumor ablation. Furthermore, to explore the dark toxicity of CP1-NCs, the hematoxylin and eosin (H&E) analysis of the tumor and major organs (heart, liver, spleen, lung, and kidney) acquired from the mice was measured after therapy. The pathomorphology analysis indicated similar morphological properties with negligible cell and tissue damage in the major organs for all mice (Figure 6(e)). The results validated that CP1-NCs could not generate significant photodamage for normal organs, although they might accumulated there.

**Figure 7:**
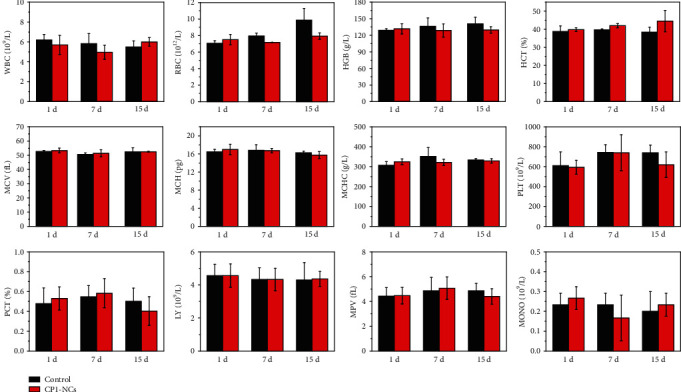
Blood hematology analysis from healthy and CP1-NC-treated mice performed at 1, 7, and 15 days. The hematology indicators include white blood cell (WBC), red blood cell (RBC), hemoglobin (HGB), hematocrit (HCT), mean corpuscular volume (MCV), mean corpuscular hemoglobin (MCH), mean corpuscular hemoglobin concentration (MCHC), platelet (PLT), plateletcrit (PCT), lymphocyte (LY), mean platelet volume (MPV), and monocyte count (MONO). Error bars correspond to standard deviation for *n* = 3.

To explore the biological toxicity of CP1-NCs, blood routine analysis and blood biochemistry were carried out. No significant changes of the blood biochemistry (Figure S12) and blood hematology (Figure 7) during the phototherapy process were found, suggesting that the CP1-NCs possess excellent biocompatibility.

## 3. Conclusion

In summary, we reported a new photothermally responsive conjugated polymeric ^1^O_2_ carrier for phase change-controlled and luminescence imaging-guided sustainable phototherapy for hypoxic tumor. The CP1-NCs not only can serve as NIR luminescence imaging agent for real-time identification of the nanocarrier accumulation but also can trap the ^1^O_2_ produced from CP1 under irradiation and form a stable EPO. The EPO undergoes cycloreversion to release ^1^O_2_ through the NIR-triggered photothermal efficiency of CP1 and controlled phase change of PCM, which can be used for oxygen-independent PDT for relieving tumor hypoxic microenvironment. As compared with CP1-NCs plus VC, the CP1-NCs exhibit better efficiency in inhibiting tumor growth through 1O2 release for enhanced cancer phototherapy. Therefore, the present study demonstrates the potential of CP1-NCs in controlled and sustainable cancer phototherapy under hypoxia, affording a new strategy for developing oxygen-independent cancer phototherapy platform.

## 4. Materials and Methods

### 4.1. Materials

Unless otherwise stated, all raw materials were bought and utilized as received. Oleic acid and 1-hexadecanol were obtained from Aldrich chemistry. The biocompatible *α*-lecithin and amphiphilic DSPE-mPEG_5000_ were purchased from Adamas-Beta. Calcein AM/PI stain kit was obtained from Nanjing KeyGen Biotech Co., Ltd. The detailed synthesis of intermediates and CPs can be found in the Supporting Information. Oleic acid and 1-hexadecanol were weighed at the various mass ratios (OA : Hex = 1 : 2.5, 3.0, 3.5, 4.0, and 4.5) and then dissolved in ethanol. Then, the mixture solution was ultrasonically mixed and stored in a refrigerator as PCM with controllable melting point.

### 4.2. Preparation and Characterization of CP-NPs

The CP-NPs were obtained through a resolidification method. The CPs and PCM (2 mg/mL) dissolved in THF solution serve as solution 1. The DSPE-mPEG_5000_ and *α*-lecithin (10 mg in 10 mL water) dispersed in aqueous solution were used as solution 2. Then, the two solutions were mixed under continuous sonication at 50°C for 6 min and then rapidly cooled in an ice bath. After the solution was warmed up to room temperature, THF was removed under stirring. The solution was filtered via a polyethersulfone (PES) syringe-driven filter (0.44 *μ*m) and was further centrifuged utilizing a centrifugal filter. The concentration of CP-NPs was determined according to absorption spectra. The morphology and particle size of CP-NPs were measured by transmission electron microscopy and dynamic light scattering.

### 4.3. Singlet Oxygen Test

To explore the singlet oxygen production and photodynamic improvement of CP-NPs, DPBF served as an ^1^O_2_ indicator. CP-NPs at different concentrations were mixed with DPBF in water solution under continuous stirring. Under 690 nm irradiation, the absorption spectra of DPBF were acquired.

### 4.4. Photothermal Performance

The temperature elevation of CP-NCs at different concentrations (75, 150, and 300 *μ*g mL^−1^) under 690 nm irradiation (0.5 W cm^−2^, 6 min) was recorded by the FLIR E40. To acquire the photothermal conversion efficiency, CP1-NCs were exposed under 690 nm light irradiation at 0.5 W cm^−2^. After the temperature reached a plateau, the light irradiation was removed for cooling down to ambient temperature.

### 4.5. Cellular Experiment

The cells were treated in Dulbecco's modified Eagle medium and provided with 10% fetal bovine serum (FBS) at 37°C with 5% CO_2_. To evaluate the cellular uptake, HeLa cells were incubated with CP1-NCs in the dark for 6 h. Then, the cells were washed with fresh PBS twice before being imaged by confocal imaging. To test the phototoxicity of CP1-NCs, HeLa cells were treated with PBS or CP1-NCs under 21% or 5% oxygen concentration for 24 h, followed by 5 min light irradiation under 690 nm (0.5 W cm^−2^). The HeLa cells were incubated with DCFH-DA at 37°C under 21% or 5% oxygen level for 30 min, followed by 690 nm irradiation at 0.5 W cm^−2^ for 5 min. Furthermore, to explore the cell population at different stages of apoptosis, AM/PI and flow cytometry assay was carried out under normoxia and hypoxia.

### 4.6. *In Vivo* Fluorescence Imaging

For *in vivo* luminescence imaging, HeLa tumor-bearing mice were intravenously injected with CP1-NCs. The luminescence signals of the mice were collected (*λ*_ex_ = 690 nm) through an IVIS Lumina K *in vivo* imaging system (PerkinElmer) using a xenon lamp which was equipped with different long- and band-pass filters. An Andor EMCCD-DU897 camera was used as an imaging detector. Images were taken at 2, 4, 8, 12, and 24 h postinjection. After 24 h, the tumor and major organs were collected and the fluorescence intensity was analyzed to confirm the accurate therapeutic time.

### 4.7. Blood Routine Examination

The whole blood was acquired from the orbital venous plexus on 1-, 7-, and 15-day postinjection and subject to blood routine test. The blood samples were acquired from the mouse fundus artery in each group. Blood solutions (100 *μ*L) were treated with anticoagulant for hematology analysis. After being kept at 4°C for 4 h and centrifuged, blood plasma samples (200 *μ*L) were obtained from blood for biochemistry assay.

### 4.8. *In Vivo* Synergistic Therapy

All tumor nude mice were acquired from Jiangsu KeyGen Biotech Co., Ltd. and used referring to the standard of the Laboratory Animal Center of Jiangsu KeyGen Biotech Co., Ltd. When the tumor volume increased to around 120 mm^3^, we began to carry out the phototherapy experiments. To assess the phototherapy effect of CP1-NCs, the tumor-bearing HeLa mice were injected with CP1-NCs at a dose of 0.9 mg kg^−1^. The tumors were exposed under 690 nm light irradiation at 0.5 W cm^−2^ for 6 min.

### 4.9. Histological Staining

After treatment, the final tumor and major organs were fixed with 4% formaldehyde for H&E staining to explore the side effect of CP1-NCs.

## Supplementary Materials

Supplementary Materials Figure S1: synthetic routes of the 1-7. Figure S2: synthetic routes of the CP1-CP3. Figure S3: morphological changes of the CP-NCs before and after NIR irradiation. The size of the CP1-NCs@PCM became smaller after the irradiation, while no obvious changes were obtained from the CP2-NPs@PCM. Scale bars: 200 nm. Figure S4: (a) UV-vis-NIR absorption spectra of CP1 at various concentrations in CH_2_Cl_2_. (b) The equation was calculated according to the maximal absorption of (a). (c) UV-vis-NIR absorption spectra of CP2 at various concentrations in CH_2_Cl_2_. (d) The equation was calculated according to the maximal absorption of (c). (e) UV-vis-NIR absorption spectra of CP3 at various concentrations in CH_2_Cl_2_. (f) The equation was calculated according to the maximal absorption of (e). Figure S5: absorption spectra of 7, CPs, and CP-NPs. Figure S6: emission spectra of CP1-CP3 in CH_2_Cl_2_ and CP-NPs in water. Figure S7: (a) absorption changes of DPBF with CP1-NCs under 690 nm irradiation in water and (inset) the absorption decrease owing to the endoperoxide formation. (b) ΔAbs of DPBF in mixture solution of CP3-NPs and DBPF. Figure S8: (a) photothermal stability of CP1-NCs. (b) The heating curve of the CP1-NCs in a procedure of laser-on and laser-off. (c) The linear cooling time data versus −ln(*θ*) acquired from the cooling period of (b). (d) Temperature elevation of CP2-NPs. Figure S9: confocal images of the cellular uptake of CP1-NCs and DAPI. Scale bar: 20 nm. Figure S10: flow cytometry quantification of annexin V-FITC and PI-labeled HeLa cells cultured with only PBS or laser, respectively. Figure S11: the CP1-NC distribution in the tumor and major organs after 12 h intravenous injection. Figure S12: blood biochemical assay. (a) Glutamic-pyruvic transaminase (ALT), (b) glutamic oxalacetic transaminase (AST), (c) urea nitrogen (BUN), and (d) creatinine (CREA) concentrations for liver and kidney functions of healthy nude mice 15 days after tail intravenous injection of CP1-NCs. Error bars represent the standard deviations (*n* = 3). Figure S13: the ^1^H NMR spectrum of 6 in CDCl_3_. Figure S14: the ^13^C NMR spectrum of 6 in CDCl_3_. Figure S15: the ^1^H NMR spectrum of 7 in CDCl_3_. Figure S16: the ^1^H NMR spectra of CP1-CP3. Figure S17: MALDI-TOF-MS spectra of 4-6.
