# Supplementary material for: Photothermally Responsive Conjugated Polymeric Singlet Oxygen Carrier for Phase Change-Controlled and Sustainable Phototherapy for Hypoxic Tumor
Source: Research (Wash D C). 2020 Oct 10;2020:5351848. doi: 10.34133/2020/5351848 (PMC7569507; doi:10.34133/2020/5351848)
Supplement: Supplementary Materials — Figure S1: synthetic routes of the 1-7. Figure S2: synthetic routes of the CP1-CP3. Figure S3: morphological changes of the CP-NCs before and after NIR irradiation. The size of the CP1-NCs@PCM became smaller after the irradiation, while no obvious changes were obtained from the CP2-NPs@PCM. Scale bars: 200 nm. Figure S4: (a) UV-vis-NIR absorption spectra of CP1 at various concentrations in CH2Cl2. (b) The equation was calculated according to the maximal absorption of (a). (c) UV-vis-NIR absorption spectra of CP2 at various concentrations in CH2Cl2. (d) The equation was calculated according to the maximal absorption of (c). (e) UV-vis-NIR absorption spectra of CP3 at various concentrations in CH2Cl2. (f) The equation was calculated according to the maximal absorption of (e). Figure S5: absorption spectra of 7, CPs, and CP-NPs. Figure S6: emission spectra of CP1-CP3 in CH2Cl2 and CP-NPs in water. Figure S7: (a) absorption changes of DPBF with CP1-NCs under 690 nm irradiation in water and (inset) the absorption decrease owing to the endoperoxide formation. (b) ΔAbs of DPBF in mixture solution of CP3-NPs and DBPF. Figure S8: (a) photothermal stability of CP1-NCs. (b) The heating curve of the CP1-NCs in a procedure of laser-on and laser-off. (c) The linear cooling time data versus −ln(θ) acquired from the cooling period of (b). (d) Temperature elevation of CP2-NPs. Figure S9: confocal images of the cellular uptake of CP1-NCs and DAPI. Scale bar: 20 nm. Figure S10: flow cytometry quantification of annexin V-FITC and PI-labeled HeLa cells cultured with only PBS or laser, respectively. Figure S11: the CP1-NC distribution in the tumor and major organs after 12 h intravenous injection. Figure S12: blood biochemical assay. (a) Glutamic-pyruvic transaminase (ALT), (b) glutamic oxalacetic transaminase (AST), (c) urea nitrogen (BUN), and (d) creatinine (CREA) concentrations for liver and kidney functions of healthy nude mice 15 days after tail intravenous injection of CP1-NCs. Er [file 5351848.f1.docx]

**Supplementary Materials**

**Photothermally-Responsive Conjugated Polymeric Singlet Oxygen Carrier for Phase-Change-Controlled and Sustainable Phototherapy for Hypoxic Tumor**

Guo Li,^1^ Ruyi Zhou,^1^ Weili Zhao,^1^ Bo Yu,^1^ Jie Zhou,^1^ Shujuan Liu,^1^* Wei Huang,^1,2^* and Qiang Zhao^1^*

^1^Key Laboratory for Organic Electronics and Information Displays & Jiangsu Key Laboratory for Biosensors, Institute of Advanced Materials (IAM), Nanjing University of Posts and Telecommunications (NUPT), 9 Wenyuan Road, Nanjing 210023, Jiangsu, P. R. China.

^2^Frontiers Science Center for Flexible Electronics (FSCFE) & Shaanxi Institute of Flexible Electronics (SIFE), Northwestern Polytechnical University (NPU), 127 West Youyi Road, Xi'an 710072, Shaanxi, P. R. China.

Correspondence should be addressed to Qiang Zhao; iamqzhao@njupt.edu.cn, Shujuan Liu; iamsjliu@njupt.edu.cn, and Wei Huang; provost@nwpu.edu.cn.

**Figure S1.** Synthetic routes of the **1-7**.

**Figure S2.** Synthetic routes of the **CP1-CP3**.

**Synthesis**

The intermediates 1 and 2 were prepared according to the reported literature [1, 2].

Synthesis and characterization of **3**: To a solution of 2,4-dimethylpyrrole (0.418 g, 4.4 mmol) and **2** (0.52 g, 2 mmol) in distilled CH_2_Cl_2_ (20 mL) was added a solution of trifluoroacetic acid (50 μL) in distilled CH_2_Cl_2_ (20 mL) slowly at room temperature. After 1 h of stirring under ice bath, 2,3-dichloro-5,6-dicyano-1,4-benzoquinone (DDQ) (0.454 g, 2 mmol) was added and stirred for additional 1 h at room temperature. Then, triethylamine (NEt_3_) (5 mL, 36 mmol) was added, followed by slow addition of boron trifluoride diethyl etherate (BF_3_·Et_2_O) (2.5 mL, 20 mmol). The reaction mixture was washed after 2 h stirring at room temperature with saturated Na_2_CO_3_ aqueous solution, dried over anhydrous Na_2_SO_4_ and the solvent was removed by rotary evaporation. The residue was purified by column chromatography over silica gel (PE/CH_2_Cl_2_, 2:1). The product was dried to yield a red-orange solid (0.56 g, 59%).^1^H NMR (CDCl_3_, 400 MHz): δ 8.20-8.15 (m, 1H), 8.12-8.07 (m, 1H), 7.65-7.58 (m, 2H), 7.55-7.50 (m, 2H), 7.42-7.37 (m, 2H), 7.30 (s, 1H), 6.05 (s, 2H), 2.75 (s, 3H), 2.60 (s, 9H), 1.56 (s, 6H) ppm.

Synthesis and characterization of **4**: **3** (0.956 g, 2 mmol) and N-Iodosuccinimide (NIS) (1.12 g, 5 mmol) were dissolved in CH_3_COOH (10 mL) and CHCl_3_ (30 mL). After the addition was complete, the solution was stirred 0.5 h at room temperature. The mixture was added anhydrous Na_2_CO_3_ and filtered. Then the mixture was extracted with dichloromethane and water, the organic layer was dried with anhydrous Na_2_SO_4_ and the solvent was removed by rotary evaporation. The residue was purified by column chromatography on silica gel eluting with petroleum ether/CH_2_Cl_2_ = 3:1. Eluent was removed by rotary evaporation to give red power (1.358 g, 93%). ^1^H NMR (CDCl_3_, 400 MHz): 8.20-8.14 (m, 1H), 8.13-8.07 (m, 1H), 7.68-7.58 (m, 2H), 7.58-7.53 (m, 2H), 7.40-7.34 (m, 2H), 7.30 (s, 1H), 2.76 (s, 3H), 2.70 (s, 6H), 2.60 (s, 3H), 1.58 (s, 6H) ppm.

Synthesis and characterization of **5**: **4** (0.73 g, 1 mmol) was dissolved in 20 mL of toluene before 40 mL of dry trimethylamine was added under N_2_ atmosphere. Pd(PPh_3_)_4_ (34.7 mg, 0.03 mmol) was added, followed by CuI (38 mg, 0.2 mmol). Trimethylsilylacetylene (78.4 mg, 0.8 mmol) was injected before the mixture was stirred for overnight. After the reaction was finished, the solvent was removed by rotary evaporation. The residue was purified by column chromatography on silica gel eluting with petroleum ether/CH_2_Cl_2_ = 4:1. **5** was collected as a dark red solid (423 mg, 63%). ^1^H NMR (CDCl_3_, 400 MHz): 8.19-8.14 (m, 1H), 8.12-8.07 (m, 1H), 7.67-7.59 (m, 2H), 7.57-7.52 (m, 2H), 7.38-7.33 (m, 2H), 7.29 (s, 1H), 2.76 (s, 3H), 2.68 (s, 6H), 2.60 (s, 3H), 1.57 (s, 6H), 0.24 (s, 18H) ppm.

The precursor **6** and **7** were prepared in the same way. The detailed synthesis of **6** is present as an example.

Synthesis and characterization **6**: **5** (0.336 g, 0.5 mmol) was dissolved in 10 mL of distilled THF. Tetrabutylammonium fluoride (1 M in THF, 2.0 mL) was added dropwise at -78 °C under nitrogen. The solution was kept at -78 °C for 1 h. After the reaction was finished, 10 mL water and 20 mL CH_2_Cl_2_ were added. The aqueous layer was extracted with CH_2_Cl_2_ (3×10 mL). The combined organic layers were dried over anhydrous Na_2_SO_4_ and the solvent was removed by reduced pressure. The residue was purified by column chromatography on silica gel eluting with petroleum ether/CH_2_Cl_2_ = 1:1. The precursor **6** was collected as a dark red solid (yield: 52%). ^1^H NMR (CDCl_3_, 400 MHz): 8.19-8.15 (m, 1H), 8.12-8.08 (m, 1H), 7.66-7.60 (m, 2H), 7.58-7.54 (m, 2H), 7.39-7.34 (m, 2H), 7.30 (s, 1H), 3.36 (s, 2H), 2.76 (s, 3H), 2.70 (s, 6H), 2.60 (s, 3H), 1.58 (s, 6H) ppm. ^13^C NMR (100 MHz, CDCl_3_) δ: 158.91, 145.54, 144.30, 143.16, 137.57, 133.04, 132.98, 132.63, 132.19, 130.78, 128.59, 127.50, 126.14, 125.67, 125.13, 124.68, 75.95, 84.21, 19.34, 16.16, 13.44 ppm.

**7** : ^1^H NMR (CDCl_3_, 400 MHz): 7.52 (t, *J* = 8.8 Hz, 3H), 7.24 (d, *J* = 8.0 Hz, 2 H), 3.31 (s, 2H), 2.65 (s, 6H), 1.46 (s, 6H) ppm.

Synthesis of **CP1**: The precursor **6** (105.2 mg, 0.2 mmol), *trans*-[PtCl_2_(P(C_4_H_9_)_3_)_2_] (**Pt1**, 134 mg, 0.2 mmol), CuI (7.6 mg, 0.04 mmol) and Et_3_N/CH_2_Cl_2_ (10 mL, 1:2, v/v) were charged in a pressure vessel under nitrogen. After stirring at room temperature overnight, the solvent was removed by rotary evaporation. The residue was purified by column chromatography. After concentration of the chloroform solution, brown solid was collected by precipitation into methanol. Subsequent washing with methanol gave the polymers as brown solid (Yield: 57.4%). ^1^H NMR (CDCl_3_, 400 MHz): 8.18-8.13 (1H, Ar-H of 1,4-dimethylnaphthalene), 8.11-8.07 (1H, ArH of 1,4-dimethylnaphthalene), 7.65-7.58 (2H, ArH of 1,4-dimethylnaphthalene), 7.52-7.47 (2H, ArH of BODIPY), 7.40-7.33 (2H, ArH of BODIPY), 7.31 (1H, ArH of 1,4-dimethylnaphthalene), 2.75 (3H, -CH_3_ of 1,4-dimethylnaphthalene), 2.66 (6H, -CH_3_ of BODIPY), 2.58 (3H, -CH_3_ of 1,4-dimethylnaphthalene), 1.62-1.54 (24H, -CH_3_ of BODIPY and side chain of Bu_3_), 1.42-1.35 (12H, side chain of Bu_3_), 0.94-0.80 (24H, side chain of Bu_3_) ppm.

Synthesis of **CP2**: A mixture of **6** (105.2 mg, 0.2 mmol), 1, 4-diiobenzene (66 mg, 0.2 mmol), Pd(PPh_3_)_4_ (11.6 mg, 0.01 mmol) and CuI (7.6 mg, 0.04 mmol) were dissolved in distilled toluene (5 mL).The dry trimethylamine (10 mL) was added into the mixture under N_2_ atmosphere. The resultant mixture was refluxed for 48 h. After the reaction was finished, the mixture was filtered and the combined organic layer was dried over anhydrous Na_2_SO_4_. The solvent was removed by rotary evaporation. Then, the mixture was poured into a large excess of methanol. The precipitate was collected and washed with hexane (Yield: 65.3%). ^1^H NMR (CDCl_3_, 400 MHz): 8.20-8.15 (1H, Ar-H of 1,4-dimethylnaphthalene), 8.13-8.07 (1H, Ar-H of 1,4-dimethylnaphthalene), 7.72-7.66 (2H, Ar-H of 1,4-dimethylnaphthalene), 7.64-7.60 (1H, Ar-H of 1,4-diiobenzene), 7.59-7.54 (2H, ArH of BODIPY), 7.45-7.38 (2H, ArH of BODIPY), 7.32-7.30 (1H, Ar-H of 1,4-diiobenzene), 7.24-7.18 (2H, Ar-H of 1,4-diiobenzene), 2.82-2.59 (12H, -CH_3_ of BODIPY and 1,4-dimethylnaphthalene), 1.74-1.68 (6H, -CH_3_ of BODIPY) ppm.

Synthesis of **CP3**: The **CP3** (Yield: 52.6%) was prepared according to **CP1** synthesis. ^1^H NMR (CDCl_3_, 400 MHz): 7.49-7.42 (3H, ArH of BODIPY), 7.25-7.22 (2H, ArH of BODIPY), 2.65-2.53 (6H, -CH_3_ of BODIPY), 1.53-1.44 (16H, -CH_3_ of BODIPY and side chain of Bu_3_), 1.39-1.28 (20H, side chain of Bu_3_), 0.94-0.81 (24H, side chain of Bu_3_) ppm.

**Figure S3.** Morphological changes of the CP-NCs before and after NIR irradiation. The size of the CP1-NCs@PCM became smaller after the irradiation while no obvious changes were obtained from the CP2-NPs@PCM. Scale bars: 200 nm.

**Figure S4.** (a) UV-vis-NIR absorption spectra of **CP1** at various concentrations in CH_2_Cl_2_. (b) The equation was calculated according to the maximal absorption of (a). (c) UV-vis-NIR absorption spectra of **CP2** at various concentrations in CH_2_Cl_2_. (d) The equation was calculated according to the maximal absorption of (c). (e) UV-vis-NIR absorption spectra of **CP3** at various concentrations in CH_2_Cl_2_. (f) The equation was calculated according to the maximal absorption of (e).

**Figure S5.** Absorption spectra of **7**, CPs and CP-NPs.

**Figure S6.** Emission spectra of **CP1**-**CP3** in CH_2_Cl_2_ and CP-NPs in water.

**Figure S7.** (a) Absorption changes of DPBF with CP1-NCs under 690 nm irradiation in water and (inset) the absorption decrease owing to the endoperoxide formation. (b) ΔAbs of DPBF in mixture solution of CP3-NPs and DBPF.

**Figure S8.** (a) Photothermal stability of CP1-NCs. (b) The heating curve of the CP1-NCs in a procedure of laser-on and off (water was served as a control). (c) The linear cooling time data versus -ln(θ) obtained from the cooling period of (b). (d) Temperature elevation of CP2-NPs.

**Figure S9**. Confocal images of the cellular uptake of CP1-NCs and DAPI. Scale bar: 20 nm.

**Figure S10.** Flow cytometry quantification of annexin V-FITC and PI-labeled HeLa cells treated with only PBS or laser, respectively.

**Figure S11.** The CP1-NCs distribution in tumor and major organs after 12 h intravenous injection.

**Figure S12.** Blood biochemical assay. (a) Glutamic-pyruvic transaminase (ALT), (b) glutamic oxalacetic transaminase (AST), (c) urea nitrogen (BUN) and (d) creatinine (CREA) concentrations for liver and kidney functions of healthy nude mice 15 days after tail intravenous injection of CP1-NCs. Error bars represent the standard deviations (n = 3).

**Figure S13.** The ^1^H NMR spectrum of **6** in CDCl_3_.

**Figure S14.** The ^13^C NMR spectrum of **6** in CDCl_3_.

**Figure S15.** The ^1^H NMR spectrum of **7** in CDCl_3_.

**Figure S16.** The ^1^H NMR spectra of **CP1-CP3**.

**Figure S17.** MALDI-TOF-MS spectra of **4**-**6**.

**References:**

[1] T. Huang, M. Zhao, Q. Yu et al., “De novo design of polymeric carrier to photothermally release singlet oxygen for hypoxic tumor treatment,” *Research*, vol. 2019, article 9269081, 11 pages, 2019.

[2] T. Huang, X. Tong, Q. Yu et al., “A series of iridophosphors with tunable excited states for hypoxia monitoring via time-resolved luminescence microscopy,” *Journal of Materials Chemistry C*, vol. 4, no. 45, pp. 10638-10645, 2016.
